# Death from Eating Cloves

**Published:** 1854-06

**Authors:** 


DEATH FROM EATING CLOVES.—Mr Amos Brown, an esteemed citizen of our village, says the *Granville Advocate*, died in convulsions, and a subsequent *post-mortem* examination showed conclusively that his death was caused by eating cloves, which he had been in the habit of using as a substitute for tobacco.

